# Public health impact and cost effectiveness of routine childhood vaccination for hepatitis a in Jordan: a dynamic model approach

**DOI:** 10.1186/s12879-018-3034-8

**Published:** 2018-03-07

**Authors:** Wail A. Hayajneh, Vincent J. Daniels, Cerise K. James, Muhammet Nabi Kanıbir, Matthew Pilsbury, Morgan Marks, Michelle G. Goveia, Elamin H. Elbasha, Erik Dasbach, Camilo J. Acosta

**Affiliations:** 1 Department of Pediatrics, Faculty of Medicine, Jordan University of Science and Technology, PO Box 3030, Irbid, 22110 Jordan; 2 Merck & Co., Inc., Kenilworth, NJ USA; 3 Agile-1 for Merck & Co., Inc., Kenilworth, NJ USA

**Keywords:** Immunization, Hepatitis a, Jordan, Impact, Cost-effectiveness analysis, Simulation, Health technology assessment, Economics

## Abstract

**Background:**

As the socioeconomic conditions in Jordan have improved over recent decades the disease and economic burden of Hepatitis A has increased. The purpose of this study is to assess the potential health and economic impact of a two-dose hepatitis A vaccine program covering one-year old children in Jordan.

**Methods:**

We adapted an age-structured population model of hepatitis A transmission dynamics to project the epidemiologic and economic impact of vaccinating one-year old children for 50 years in Jordan. The epidemiologic model was calibrated using local data on hepatitis A in Jordan. These data included seroprevalence and incidence data from the Jordan Ministry of Health as well as hospitalization data from King Abdullah University Hospital in Irbid, Jordan. We assumed 90% of all children would be vaccinated with the two-dose regimen by two years of age. The economic evaluation adopted a societal perspective and measured benefits using the quality-adjusted life-year (QALY).

**Results:**

The modeled vaccination program reduced the incidence of hepatitis A in Jordan by 99%, 50 years after its introduction. The model projected 4.26 million avoided hepatitis A infections, 1.42 million outpatient visits, 22,475 hospitalizations, 508 fulminant cases, 95 liver transplants, and 76 deaths over a 50 year time horizon. In addition, we found, over a 50 year time horizon, the vaccination program would gain 37,502 QALYs and save over $42.6 million in total costs. The vaccination program became cost-saving within 6 years of its introduction and was highly cost-effective during the first 5 years.

**Conclusion:**

A vaccination program covering one-year old children is projected to be a cost-saving intervention that will significantly reduce the public health and economic burden of hepatitis A in Jordan.

**Electronic supplementary material:**

The online version of this article (10.1186/s12879-018-3034-8) contains supplementary material, which is available to authorized users.

## Background

Hepatitis A virus (HAV) infections continue to pose significant public health issues in Jordan. Some studies over the past few decades had placed Jordan among countries with high HAV endemicity [1–3]. High endemicity is defined as seroprevalence ≥90% by age 10 years and intermediate endemicity is defined as ≥50% by age 15 years [4]. However, the most recent study conducted in 2008 [5], found that a shift in HAV infection burden from children to young adults, that is, the number of children acquiring the infection has decreased while the number of infections in adolescents and adults has increased. This epidemiological pattern among older and relatively active cohorts may lead to serious outbreaks and significant burden of disease because HAV infection in older patients is usually more symptomatic.

The World Health Organization (WHO) recommends including hepatitis A vaccination in the national immunization schedule for children ≥1 year of age when indicated, based on: incidence of acute hepatitis A; change in endemicity from high to intermediate; and consideration of cost effectiveness. According to WHO guidelines, in countries transitioning to intermediate-endemicity where the majority of the adult population will likely be susceptible to hepatitis A infection, large-scale hepatitis A vaccination programs are likely to be cost-effective and are encouraged [6].

The National Immunization Technical Advisory Group (NITAG) of Jordan has voted to introduce several vaccines: pneumococcal conjugate, rotavirus, hepatitis A and varicella to the National Immunization Program. As of December 2015, only rotavirus was introduced. In view of financial and logistic obstacles facing new vaccines introduction, cost-effectiveness and disease burden studies are expected to help decision and policymakers determine prioritization of remaining in-plan vaccines including the hepatitis A vaccine in Jordan. The purpose of this paper is to test this evidence by assessing the potential health and economic impact of a hepatitis A vaccination program covering one-year old children in Jordan.

## Methods

### Modeling approach

**Fig. 1:**
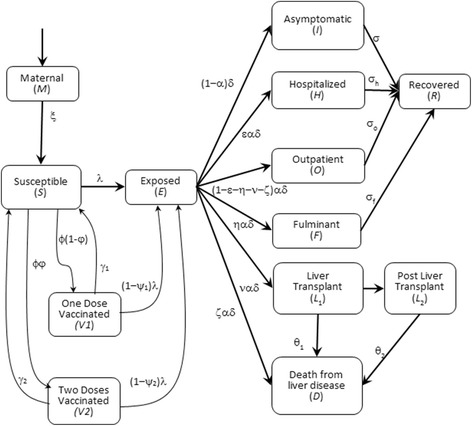
Transmission model. Flow diagram of hepatitis A virus (HAV) transmission and vaccination model: newborns enter age group a = 0, and are assumed to be protected by maternal antibodies (*M*), the protection is lost over time so the children become susceptible (*S*). Upon infection, a person moves to the exposed compartment (*E*) becomes infectious after a latent period. The model distinguishes between several categories of infection and disease: Asymptomatic (*I*), Symptomatic infections are treated as outpatient (*O*), hospitalized (*H*), with fulminant disease (*F*), requiring a liver transplant (*L*), or die from Hepatitis A virus infection (*D*). Infected persons can clear their infection and move to the recovered compartment (*R*) with lifelong immunity. Susceptible individuals may be vaccinated, one dose or two and move to the appropriate Vaccinated (V1 or V2) compartment. Vaccinated individuals may move back to the susceptible (S) compartment due to waning vaccine or to the exposed compartment due to breakthrough infections. The model also applied age-specific all-cause mortality (not shown) to all persons in all epidemiologic classes

To assess the potential health and economic impact of a two-dose hepatitis A vaccination program targeting one-year-old children in Jordan, we used a deterministic, age-structured, epidemiologic dynamic model developed previously by Dhankhar, et al. [7] and adapted for the study of HAV transmission and vaccination against HAV infection in Jordan. The model divides the population of Jordan into several distinct classes: maternal antibodies, susceptible, exposed, asymptomatic infection, outpatient, hospitalized, fulminant, patients with recent liver transplant, patients with past liver transplant, recovered, and vaccinated (Fig. 1). Each compartment was further categorized into 101 age groups (0 to < 1, 1,.. .,99, and 100 years or older). The movement of populations between compartements in the model is described in detail in Dhankhar et al. [7]

The model used in the simulations consists of 1313 coupled differential equations corresponding to the 12 epidemiological classes (see Fig. 1) and demographic equations for 101 age groups. All differential equations and details of the epidemiologic model are given in the technical supplement.

### Vaccination strategies

Jordan does not have a national HAV vaccination program in place. Also, we were unable to find any data indicating signficant HAV vaccination through the private health sector. Hence, we assumed that before the start of universal vaccination no hepatitis A vaccination strategy was in place in Jordan and that, for our base case, a two-dose program covering oneyear-olds began in 2016. The program consisted of one dose at 12 months old and the second dose at 18 months old. .

In the model, the vaccination at age one year is incorporated by moving a fraction of infants from the susceptible class to the vaccination class before they are two years old. We compared routine universal vaccination of one year-olds to a no vaccine strategy.

### Model parameters and data sources

We identified base case inputvalues of demographic, epidemiologic, vaccine, and economic inputs of the model through an extensive search of the published literature. The resulting input values are detailed in Table 1. Jordan specific data inputs were also identified and used where they were available.

**Table 1:** Model parameters

Parameter	Base Case	Range	Distribution	Reference
Demography				
Population 2014	8,117,564	Fixed		[10]
Age Specific All-cause Mortality	Varies	Fixed		[8]
Infection				
Force of Infection		Fixed		[20, 44]
Scale parameter	0.854327	Fixed		
Shape parameter	0.00159689	Fixed		
Shape parameter	11.5766	Fixed		
Duration of Maternal Immunity (mo)	6.5	Fixed		[45]
Latency period (d)	14	Fixed		[46, 47]
Age-specific mean duration of infectiousness (wk)		Fixed		[48, 49]
0–4 y	3.5			
5–9 y	3.0			
10+ y	2.5			
Vaccine				
Vaccine efficacy, one does only (%)	100	87.3–100	Derived Distribution	[22]
Vaccine efficacy, two doses (%)	100	87.3–100	Derived Distribution	Assumed
Median duration of vaccine-derived immunity, one dose only (y)	21	20.3–21.9	Gamma (2647.1, 0.0079)	Assumed
Median duration of vaccine-derived immunity, two doses (y)	32	30.9–33.4	Gamma (2517.63,0.127)	[23]
Vaccination Strategy				
Vaccine uptake, universal strategy, first dose (%)	95%	80–97	Beta (34.37426,1.0007488)	Assumed
Vaccine adherence, probability of second dose given first dose (%)	95%	70–95	Beta (27.15759,2.49654)	Assumed
Clinical Outcomes				
Under reporting factor	11.76	Fixed		Estimated
Probability an infection is icteric	Varies	Fixed		[20]
Proportion of icteric infections hospitalized (%)				[21]
≤ 4 y	0.395		Dirichlet(D1)^a^	
5–14 y	1.45		Dirichlet(D2)	
15–39 y	1.91		Dirichlet(D3)	
40–59 y	1.57		Dirichlet(D4)	
60+ y	1.7		Dirichlet(D5)	
Probability of fulminant hepatitis A from an icteric infection (%)				[21]
≤ 4 y	0.0323		Dirichlet(D1)	
5–14 y	0.00425		Dirichlet(D2)	
15–39 y	0.0578		Dirichlet(D3)	
40–59 y	0.468		Dirichlet(D4)	
60+ y	0.68		Dirichlet(D5)	
Probability of liver transplant from an icteric infection (%)				[21]
≤ 4 y	0.00606		Dirichlet(D1)	
5–14 y	0.000797		Dirichlet(D2)	
15–39 y	0.0108		Dirichlet(D3)	
40–59 y	0.0877		Dirichlet(D4)	
60+ y	0.017		Dirichlet(D5)	
Probability of death from an icteric infection (%)				[21]
≤ 4 y	0.00468		Dirichlet(D1)	
5–14 y	0.000616		Dirichlet(D2)	
15–39 y	0.00838		Dirichlet(D3)	
40–59 y	0.0678		Dirichlet(D4)	
60+ y	0.456		Dirichlet(D5)	
Probability of death during first year of liver transplant (%)	11.6	6.0–42.0	Beta (1.14982, 8.76245)	[50]
Probability of death from year 2 and beyond after liver transplant (%)	4.4	2.4–11.0	Beta (3.80137, 82.5935)	[50]
Duration of outpatient icteric infection (d)	34.4	17–40	Gamma (34.37,1.00)	[51]
Duration of inpatient icteric infection (d)	67.8	27–78	Gamma (27.16,2.50)	[51]
Economic Parameters				
Cost of one-dose vaccine (2015 $)	10.00	8.00–14.00	Gamma (42.6844, 0.234277,1.1,3)	Estimated
Test Costs	22.55	Fixed		[26]
Cost of one-dose administration	1.91	Fixed		Estimated
outpatient cost	$15.06	10–25	Gamma (8.71228, 7.77408)	[27]
outpatient visits	3	1–4		Estimated
hospitalization cost	$ 111.5	80–150	Gamma (6.43896, 81.4262)	[26]
hospitalization duration	4.5	3–7		Estimated
fulminant cost	$35,715	32,143–39,278	Gamma (385.022, 92.7609)	[26]
annual cost of patients with liver transplant (1st year)	$70,000	50,000–90,000	Gamma (47.0596, 1487.48)	[26]
annual cost of patients with liver transplant (subsequent years)	43,203	34,563–51,843	Gamma(96.0533, 449.781)	Estimated
Public health cost of a reported infection (2013 $)	0	0–15	Gamma(1.092722,3.660584)	Estimated
work loss, outpatient (d)	15.5	7–18	Gamma(30.51056,0.508021)	[51]
work loss, inpatient (d)	33.2	10–25	Gamma(75.2776,0.4410342)	[51]
work loss, fulminant (d)	33.2	10–25	Gamma(75.2776,0.4410342)	[51]
work loss, year of transplant (d)				[25]
0–17 y	153.2	145–160	Gamma(1602.9, 0.0955766)	
18–40 y	245	238–253	Gamma (4099.41, 0.0597646)	
41–55 y	271	268–274	Gamma (31,347.9, 0.00864492)	
56–62 y	288	284–291	Gamma (26,011.2, 0.0110721)	
63+	314	306–321	Gamma (6733.62, 0.0466316)	
Labor force participation (%)				[52]
16–19 y	3.15	1–28	Beta[1.4452,44.4342]	
20–24 y	33.8	31–72	Beta[6.57547,12.8786]	
25–34 y	64.0	57–70	Beta[133.435,75.0572]	
35–44 y	59.6	51 – 71	Beta[54.5338,36.9658]	
45–54 y	47.1	35–59	Beta[30.8364,34.6337]	
55–64 y	30.7	0–62	Beta[2.30395,5.20078]	
65+	10.0	0–23	Beta[2.51432,22.6289]	
Median Monthly earnings (2014 $)				[53]
< 25 y	292	137–350	Gamma(13.6337, 21.4175)	
25+	365	250–650	Gamma(28.8788, 10.1112)	
Discount rate per year, %	3	0–5		Assumed
Utilities				
Average population norms				[31]
20–29 y	.920	0.913–0.927	Beta(5307.7008, 461.5392)	
30–39 y	.905	0.898–0.912	Beta(6099.1932,640.2468)	
40–49 y	.874	0.867–0.882	Beta(6572.4195,947.5113)	
50–59 y	.848	0.840–0.857	Beta(5810.9376,1041.5832)	
60–69 y	.824	0.812–0.836	Beta(3187.1645,680.7536)	
70–79 y	.785	0.771–0.798	Beta(2791.9027,764.6613)	
80+ y	.744	0.720–0.768	Beta(944.3511,324.9380)	
Persons with anicteric hepatitis A	.830	0.789–0.867	Beta(291.9525,59.7975)	[21]
Persons with icteric hepatitis A	.642	0.607–0.682	Beta(410.1317,228.3050)	[29]
Persons with liver transplant	.730	0.630–0.840	Beta(49.4052,18.2732)	[30]

Base-case model parameters, estimates, corresponding distributions and ranges for deterministic and probabilistic sensitivity analysis, and data source references

^a^Parameters for the Dirichlet distributions are as follows: D1 = (97.0207,0.385265,0.00456518,0.031484,0.00590325), D2 = (349.97,5.13289,0.00218903,0.0150967,0.00283064), D3 = (458.787,8.95484,0.0392421,0.270635,0.0507441), D4 = (377.566,6.07215,0.261759,1.80523,0.338481), D5 = (161.413,2.82536,0.757196,1.13014,0.0282536)

#### Demographic parameters

We obtained age and gender-specific mortality from both the WHO Life Tables [8] and Jordan’s IndexMundi demographic profile [9]. Population size of Jordan for 2014 was obtained from U.S. Census International Database [10] and includes Syrian refugee population.

#### Transmission rates and force of infection

The epidemiologic data on HAV in Jordan and the region suggest there is an ongoing shift from high endemicity to intermediate endemicity [3, 5]. The transmission of HAV in general is correlated with socioeconomic conditions and in particular sanitation and water supply infrastructure [3, 11, 12]. To account for this shift, we adjusted the age-specific force of infection by a universal (age independent) multiplier consisting of piecewise function of time that gradually reduces the force of infection. We allowed for two periods of change each at a different rate. Both the dates and the magnitude of the reduction in force of infection were determined from calibration to local data. To estimate the forces of infection before the vaccination program is initiated, we used least square methods to fit the model to data on age specific seroprevalence levels in 2008 [5] and seroprevalence in 1988 data [2]. In addition we used incidence data from Jordan Ministry of Health between 2004 and 2014 [13]. Using these data, the force of infection multiplier function indicated a 4% per year decline in force of infection between 1984 and 1999 followed by a 1% reduction for the next 10 years (see Additional file 1). The timing and magnitude of the trends are consistent with the historical socioeconmic development of Jordan as well as specific sanitation and water supply improvement efforts over the past few decades [14, 15]. Additionally, the decline in force of infections ends in about 2009 as the Syrian crisis in the region [16] begins and ultimately strains resources as a significant new population with a different level of HAV endemicity is introduced into the Jordan population [17–19].

To estimate the proportion of non-outpatient cases we used unpublished HAV hospitalization data from King Abdullah University Hospital in Jordan over the years 2003 through 2013 (see Additional file 1). The proportion of icteric HAV infections is determined using an age specific probability of developing jaundice [20].

The modeling approach does not assume that the endemic disease has reached an equilibrium state before vaccination. Thus, force of infection estimates continue to change with time during the prevaccination period. Force of infection was modeled assuming proportionate mixing, and no exogenous effects, for example, exposure during foreign travel. The force of infection is dynamically modeled so that herd effects are naturally included. Detailed description of the forces of infection are available in the supplementary material (see Additional file 1). Other parameters of the natural history of HAV infection are summarized in Table 1.

#### Hepatitis a virus infection parameters

We modeled several health outcomes associated with the HAV infection including outpatient visits, hospitalizations, fulminant hepatitis disease, liver transplant, and death (Fig. 1). We assumed that asymptomatic hepatitis A cases do not require health care and only the management of icteric hepatitis A cases entails the use of health care resources. We further assumed that all reported and unreported icteric cases are treated and used the underreporting factor to identify the proportion of inpatient – hospitalized, fulminant hepatitis, or liver transplant – the remaining cases are assumed to receive outpatient care. Within the inpatient category the conditional probabilities shown in Table 1 are the result of dividing by the underreporting factor [7, 21].

#### Vaccine parameters

Vaccine efficacy for one or two doses was based on data from VAQTA [Hepatitis A Vaccine, Purified Inactivated, Merck & Co., Inc., Kenilworth, NJ] and was determined to be 100% in the base case [22]. We assumed the median duration of protection of a completed one-dose and two-dose hepatitis A vaccination in the base case was 21 and 32 years, respectively, and examined alternative assumptions in the sensitivity analysis [23]. With respect to vaccine coverage, the base case assumed that 95% of one-year-olds will receive one-dose and that 95% of those will receive the second does before they are two years old. The base case vaccination coverage was chosen based on coverage achieved in similar childhood vaccinations in the Jordanian National Immunization Program [13, 24].

#### Costs

We adopted a societal perspective for economic evaluation. We included both direct and indirect costs in the evaluation. The direct costs included treatment costs of HAV-associated health outcomes and vaccination. Treatment costs included costs for outpatient visits, hospitalizations, fulminant hepatitis, and liver transplantations [25–27]. Wherever possible we used Jordanian cost data, however, in some cases we used costs from similarly economically situated countries and adjusted appropriately. We use $10.00 USD per dose for the vaccine price based on estimates of current regional pricing. We assumed $1.90 for vaccine administration costs based on estimates from King Abdullah University Hospital.

Indirect costs included the costs associated with the work loss due to different health outcomes. These were estimated by multiplying the days of work loss from an HAV disease outcome by the labor force participation rate and the daily wages of the patients’ age group (Table 1). We did not include public health disease control costs in the base case study as these were estimated to be very small in Jordan. However, we did include non-zero public health costs in the sensitivity analyses. All costs are reported in 2015 USD. Future costs were discounted at 3% per year. To interpret the cost-effectiveness of vaccination we used the WHO CHOICE criteria which set cost-effectiveness thresholds based on per capita GDP. According to this criteria, incremental cost-effectiveness ratio (ICER) less than one times per capita GDP is considered “very cost effective” while ICER greater than three times per capita GDP is “not cost-effective” [28].

#### Utilities

We calculated quality-adjusted life-years (QALYs) by applying utility weights to time spent in each health state. We assumed a weight of 0.642 for all icteric outcomes related to hepatitis A, except liver transplant [29]. For patients with liver transplants, we assumed a weight of 0.73 following transplantation [30]. We assumed the quality-of-life weight of 0.83 for persons with anicteric hepatitis A. For all other health states we used a weight of 1.0. To account for the age-specific health utilities in the general population, all utility weights were multiplied by the age-specific US population norms [31] due to a lack of Jordanian-specific data.

### Implementation

The model was implemented in Mathematica 10.1 (Wolfram Research, Inc., Champaign, IL). We used the built-in function NDSolve to find numerical solutions to the system of ordinary differential equations. The built-in function NMinimize was used for model calibration. We used Mathematica for all simulations, and analyses and some figures; others were produced using Microsoft Excel 2010 (Microsoft Corp., Redmond, WA).

## Analyses

### Base case and sensitivity analyses

We estimated expected health outcomes (total HAV infections, symptomatic HAV infections, outpatient visits, hospitalizations, fulminant hepatitis cases, number of liver transplants, deaths, QALYs) and costs over a time horizon of 50 years.

Deterministic sensitivity analyses were performed by varying vaccination parameters (efficacy, duration of protection, cost, coverage, and adherence), cost parameters, quality-of-life weights, and discount rate.

Probabilistic sensitivity analyses (PSA) were also performed using a subset of parameters including vaccine properties and uptake, clinical outcomes, costs, and quality-of-life weights were included in a PSA as described in Table 1. The rationale for the selection of the probability distribution function for each parameter and sampling techniques used in the PSA is discussed in the technical supplement. The results are summarized using the mean of outcomes and presented using a scatter plot in the incremental cost-effectiveness plane [32]. The location of a point on the plane summarizes its cost and effectiveness. For example, all positive incremental QALYs represent health gains; negative costs represent a total cost savings for the program. If the costs are positive and the QALYs are positive then there is a threshold, the willingness to pay (WTP) for each QALY gained below which the program is considered cost-effective or cost-saving.

### Model transparency and validation

To facilitate independent review and replication of our results according to industry recommendations [33] we provided a technical supplement which includes information on the model’s structure, equations, inputs, outputs, and detailed description of methods of the derivation. The face validity of the model is described in Dhankhar, et al. [7] Internal validity of the model code was verified using several tests including balanced inflow and outflow between compartments and constancy of total population throughout the calculations. The predictive validity of the model was evaluated by comparing model results with epidemiologic data reported in the literature (see Additional file 1).

## Results

### Base case

#### Epidemiology results

**Fig. 2:**
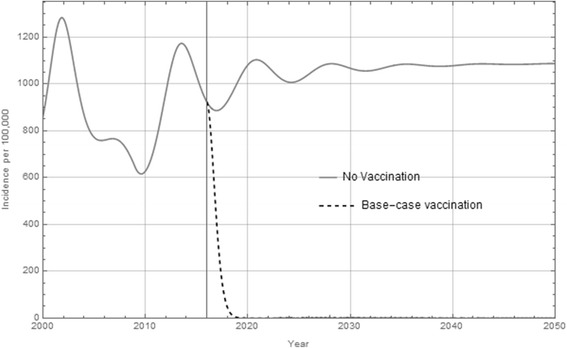
hepatitis A incidence. Overall incidence of hepatitis A with no vaccine (solid line) and after vaccination (dashed line) assuming the vaccination program begins in 2016

The model predicts rapid and substantial decrease in overall incidence of Hepatitis A (Fig. 2). The model predicts a drop from 900 cases per 100,000 a year down to less than one case per 100,000 within five years of launching the vaccination program. Long-term health outcomes were predicted after the introduction of the vaccination program (Table 2). In terms of cumulative outcomes, on the 50 year time horizon there are 4.26 million infections avoided resulting in more than 1.4 million inpatient cases avoided over the 50 year time horizon.

**Table 2:** Cumulative HAV cases avoided for the population of Jordan after 5, 10, 25, and 50 years of universal vaccination

Cumulative events avoided with vaccination	Universal Vaccination			
	5 years	10 years	25 years	50 years
Any Infection	326,900	748,608	1,618,125	4,260,589
Asymptomatic	225,772	509,773	1,085,502	2,813,184
Symptomatic	101,127	238,835	532,624	1,447,405
Outpatient	99,572	235,120	524,228	1,424,252
Hospitalizations	1522	3633	8195	22,475
Fulminant cases	24	61	151	508
Liver Transplants	5	11	28	95
Deaths	4	9	22	76

#### Cost-effectiveness results

The economic impact of the vaccination program is summarized in Table 3. The results for costs and QALYs are shown for the whole population. Costs savings are achieved by 6 years after the program begins saving $46.2 million in the first 50 years.

**Table 3:** Cumulative cost effectiveness/savings after 5, 10, 25, and 50 years of vaccination program. Cost savings is achieved within 6 years considering indirect costs and within 8 years if indirect costs are excluded. Values are for the entire population

Cumulative outcomes	Universal Vaccination			
	5 y	10 y	25 y	50 y
QALY’s Gained^a^	4800	10,521	24,144	37,502
Vaccination Cost (millions)	$9.40	$17.52	$35.76	$52.83
Disease Cost Avoided (millions)	$8.06	$18.60	$47.78	$81.41
Indirect cost avoided (millions)	$0.99	$2.61	$8.00	$14.03
Total Cost Savings(millions, including indirect costs)	–	$3.69	$20.03	$42.60
Total Cost Savings(millions, excluding indirect cost)	–	$1.09	$12.02	$28.57
ICER^b^ ($/QALY, including indirect costs)	75	Cost saving	Cost saving	Cost saving
ICER ($/QALY, excluding indirect cost)	281	Cost saving	Cost saving	Cost saving

^a^QALY, quality-adjusted life-year

^b^ICER, incremental cost-effectiveness ratio

### Deterministic sensitivity analysis

The number of years to reach cost savings was most sensitive to outpatient cost, vaccine dose cost, hospitalization costs, second dose vaccine adherence, and outpatient work-loss days. The outpatient costs parameter induced the largest change. In the low cost ($10) limit, the universal vaccination program takes more than 45 years to become cost saving while in the high cost limit ($100) cost saving is attained within 4 years. The sensitivity to this factor arises from the large proportion of cases that are treated as outpatient, approximately 99% of all symptomatic cases. The next most influential parameter was the vaccine cost. The vaccine cost lower limit ($8) leads to cost savings within 4 years while the upper limit ($14) extends time to cost savings to 14 years. This sensitivity is due to the vaccine cost being the only substantial contributor to the total cost of the vaccination program.

The other factors tested lead to between − 1.4 and 1.3 years difference in reaching the cost savings threshold. For example, the lower limit of the hospitalization costs ($80) results in 6 years to cost savings while the upper limit ($150) leads to 4.6 years to cost savings. Among factors that had little effect on years-to-cost savings were the 1st dose vaccine uptake and vaccine parameters such as vaccine efficacy and duration of protection. These likewise had little effect on health outcomes. For example, with the lowest uptake, 70%, the program still reaches less than one case per 100,000 people within the five years. In fact, as long as the 1st dose uptake is > 40% this threshold will be reached. In addition, since the population has not reached endemic equilibrium by 2016, we considered a range of vaccination program start years (t_vstart_) from 2016 through 2040. We found little qualitative difference in outcomes, for example, the time to cost savings ranged from approximately 2 to 5.4 years, with our base case, t_vstart_ = 2016, being the worst case.

The total cost savings over 50 years was most sensitive to discount rate, outpatient costs, vaccine cost, median monthly earnings 25+, probability of death after 1st year liver transplant, outpatient work loss days, and hospitalization costs in that order. Discount rate produced the largest change reducing cost savings by $9.2 million between the low (0%) and high (5%) discount rates. Outpatient costs was the second most influential parameter increasing cost savings by $7.9 million between the low and high cost values. Vaccine cost was the third most influential parameter reducing cost savings by $3.3 million between the low and high limits. All other parameters were less influential leading to changes in cost savings between -$1.3 and $1.2 million.

### Probabilistic sensitivity analysis (PSA)

We performed a PSA using 1024 Monte Carlo simulations. The results showed that the mean incremental cost savings for the population after 50 years was US $807,000 (95% confidence interval $41,000 to $2.1 M) with a median value of $560,000. The corresponding values for incremental QALYs gained was 460 (95% confidence interval 428 to 508) with a median value of 453. Additionally, the mean number of years to reach cost saving was 6.9 years (95% confidence interval 2.2 to 30 years) with a median value of 4.4 years.

**Fig. 3:**
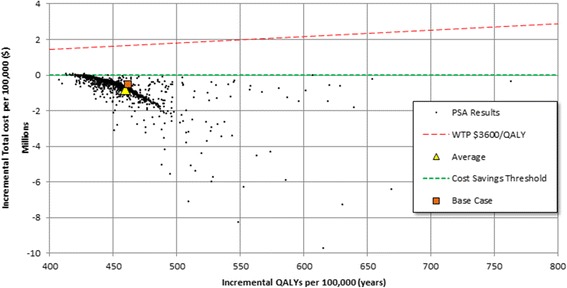
Probabilistic sensitivity analysis (PSA) results. Scatter plot of the estimated joint density of incremental total costs and QALYs per 100,000 persons for vaccination program compared to no vaccination. The long dashed line represents the $3600 WTP threshold above which the program would not be very cost effective. Each black dot represents the resulting incremental cost and effectiveness of one set of parameters from the PSA. The triangle indicates the result corresponding to the average incremental cost and effectiveness. And the square represents the cost-effectiveness of the base case parameter set. All points with negative incremental cost represent cost-saving parameter sets

The incremental cost-effectiveness plane shows that most (99.3%) of the PSA pairs fall in the fourth quadrant indicating they are cost saving (Fig. 3). The remaining 0.7% are in the first quadrant and are highly cost-effective. The average incremental cost-effectiveness ratio for the PSA pairs in the first quadrant is $86/QALY. The chart shows the line indicating a willingness-to-pay of $3600/QALY. This quantity is the one-times the per capita GDP of Jordan in 2014 below which therapies are considered “very cost-effective” according WHO CHOICE recommendations [28]. All of the scenarios in these analyses fell well below the $3600/QALY threshold.

## Discussion

To our knowledge this is the first published modeling study of HAV in Jordan. We adapted a previously published dynamic transmission model to examine the potential impact of two-dose hepatitis A vaccination of one year-olds in Jordan. As compared with no vaccination, the model predicted that this vaccination program is cost-saving within 6 years of its introduction. The model showed the virtual elimination of HAV cases within five years of introducing a two-dose vaccination program targeting one-year olds.

Our health economic results are similar to those previously obtained from model-based analysis in other HAV intermediate endemic countries. Those analyses also support the clinical and economic value of universal mass vaccination against hepatitis A virus: > 98% reduction in incidence for toddlers and > 95% reduction for the rest of the population in the first five years in Israel [34]; a sharp disease rate decrease (88%) in Argentina [35]; total disease societal cost reduction while costing the health system <US$726 per QALY in Chile [36]; cost-saving -US $ 2.26 saved for each invested dollar- in Brazil [37, 38]; and 85 to 93% reduction for all HAV infections in Mexico [39].

Sensitivity analyses showed that outpatient costs, vaccine costs, probability of death after the 1st year of a liver transplant, were most influential in determining the time to reach cost saving. No scenarios failed to reach cost saving within 50 years. Health outcomes were not particularly sensitive to uptake or adherence. Regardless, suboptimal uptake and adherence is not expected considering consistently very high uptake levels reported by Jordanian National Immunization Program with other vaccination programs [13].

Despite similarity of overall economic value with above mentioned previous studies, their results show less optimistic health outcomes than our model’s predictions [26, 36, 39, 40]. Some of the differences could be due to methodological quality variation across studies [41], but the most significant effects are likely to be due to differences in the way exogenous effects are included in the force of infection.

Our study has several limitations. First, given the lack of directly measurable data on the force of infection parameters, we had to specify a particular functional form, make simplifying assumptions, and estimate the input parameters by fitting the model to real world data. For example, when considering exogenous factors affecting the force of infection, we excluded effects due to foreign travel, or immigration. This may be a good choice for the current state, but such factors, like the ongoing influx of refugees, may become important in the future. In addition, we have assumed that ongoing and future socioeconomic changes will not have significant influence on the force of infection in the future thereby potentially overestimating the benefit of a vaccination program. Second, limited availability of reliable historical data for seroprevalence and disease incidence adds uncertainty to the model calibration. Third, we assumed that certain parameters, e.g., population size and birth rate, stay constant over the time horizon. Fourth, due to the lack of data availability for Jordan, many economic and outcomes parameters were estimated, derived, or adapted from data from other countries. Finally, we did not incorporate uncertainty in the estimated parameters of the force of infection as part of the probabilistic sensitivity analysis.

## Conclusion and recommendations

This model predicted that a universal 2-dose program vaccinating one year olds against hepatitis A led to significant reductions in hepatitis A morbidity and mortality and was cost saving within 6 years and highly cost-effective during the first 5 years.

Based on the study results, the safety profile of the vaccine [42, 43] and the WHO recommendations [6], the risk-benefit profile calls for the introduction of a two-dose program of vaccinating one-year olds against hepatitis A. These two doses, at 12 and 18 months of age, could ideally be administered simultaneously with other vaccines already included in the Jordanian National Pediatric Immunization Program.

## Additional file

Additional file 1: Technical Supplement. Modeling and analysis details. (DOCX 231 kb)

## Abbreviations

GDP: Gross domestic product
HAV: Hepatitis A virus
ICER: Incremental cost-effectiveness ratio
NITAG: National Immunization Technical Advisory Group
PSA: Probabilistic sensitivity analysis
QALY: Quality-adjusted life-year
WHO: World Health Organization
WTP: Willingness to pay
