# Correction to: Public health impact and cost effectiveness of routine childhood vaccination for hepatitis a in Jordan: a dynamic model approach

**DOI:** 10.1186/s12879-019-4533-y

**Published:** 2019-10-28

**Authors:** Wail A. Hayajneh, Vincent J. Daniels, Cerise K. James, Muhammet Nabi Kanıbir, Matthew Pilsbury, Morgan Marks, Michelle G. Goveia, Elamin H. Elbasha, Erik Dasbach, Camilo J. Acosta

**Affiliations:** 1 Department of Pediatrics, Faculty of Medicine, Jordan University of Science and Technology, PO Box 3030, Irbid, 22110 Jordan; 2 Merck & Co., Inc., Kenilworth, NJ USA; 3 Agile-1 for Merck & Co., Inc., Kenilworth, NJ USA

**Correction to: BMC Infect Dis (2018) 18(1):119**

**https://doi.org/10.1186/s12879-018-3034-8**

Following publication of the original article [1], the authors noted the following:

1. an omission of the maternally immune population from the calculation of the total vaccine cost.
2. an inaccurate representation of the Jordan population structure in the demographic model

The first issue was addressed by including all vaccinated population in the vaccine cost calculation.

The second issue was addressed by modifying the demographic portion of the model as follows. The model now assumes a constant population age distribution, a constant fertility rate, and a fixed birth cohort size consistent with 2015 Jordan population data [2–4]. To satisfy these model input assumptions we relaxed the previously required constant population size requirement to allow a constant population growth rate of 1.98% per year.

After applying these two changes to the model, the corrected base-case results continue to support the overall conclusion that the vaccination program was cost-effective assuming a willingness-to-pay of $ USD 3600 per quality-adjusted life-year (QALY) (1 x per capita Jordan gross domestic product in 2015 [5]). The updated incremental cost-effectiveness ratio is $ USD 923 per QALY. Over the 50 year analysis period, the model estimates a discounted total vaccine cost of $ USD 192.8 million; 6.2 million cumulative cases of hepatitis A infection avoided with more than 2.6 million of these being symptomatic cases. The updated probabilistic sensitivity analysis indicates all variations remain cost-effective and approximately 8% of variations will lead to cost-saving compared with no vaccination.

For updated results please see Additional files 1 and 2. Further detailed results are available from the authors upon request.

## Supplementary information

**Additional file 1.** Updated Results (Correction Results Details).

**Additional file 2.** Updated Results (Updated Tables).
