# Supplementary material for: Public health impact and cost effectiveness of routine childhood vaccination for hepatitis a in Jordan: a dynamic model approach
Source: BMC Infect Dis. 2018 Mar 7;18:119. doi: 10.1186/s12879-018-3034-8 (PMC5842652; doi:10.1186/s12879-018-3034-8)
Supplement: Supplementary file 1 — Technical Supplement. Modeling and analysis details. (DOCX 231 kb) [file 12879_2018_3034_MOESM1_ESM.docx]

An age-structured model of Hepatitis A Transmission and Vaccination in Jordan

Technical Supplement

Accompanying manuscript:

“Public Health Impact and Cost Effectiveness of Routine Childhood Vaccination for Hepatitis A in Jordan: A Dynamic Model Approach”

# Introduction

The age-structured model is divided into two major components. The first part is a description of the demographic aspects of the model. This component of the model is intended to mimic the current age structure of the Jordan population. The second part consists of the epidemiologic model that describes infection transmission in this age-structured population.

# Demographic Model

The demographic model defines the demographic characteristics of the population simulated and describes how persons enter, age, and exit the model. It is similar to the initial-boundary-value problem for age-dependent population growth described in more detail in Hethcote 1997[1]. The population is divided into *n* age groups defined by the age intervals [*a_i_*_–1_, *a_i_*], where *a*_1_< *a*_2_ < …< *a_n_* = ∞. The number of individuals *N_i_*(*t*) at time *t* in the age interval [*a_i_*_–1_, *a_i_*] is the integral of the age distribution function from *a_i_*_–1_ to *a_i_*. Assuming that the population distribution has reached a steady state with zero growth or decay, Hethcote derived a system of *n* ordinary differential equations (ODEs) for the sizes of the *n* age groups.

The simple demographic model used here divides the population into 101 age (*i* = 0,1,…,100) groups (0 to <1, 1, 2, …, 99, and 100 years or older). New additions to the population enter at rate Λ. Persons are transferred between successive age groups at an age-specific per capita rate *d_i_* per year. The transfer rate *d_i_* is given by

$$d_{i}=\frac{\mu_{i}}{e^{\left( a_{i}-a_{i-1} \right)\mu_{i}}-1}$$

The demographic model is given by the following system of 101 ODEs:

$$\frac{dN_{0}}{dt}=\Lambda-\left( \mu_{0}+d_{0} \right)N_{0}$$

$$\frac{dN_{i}}{dt}=d_{i-1}N_{i-1}-\left( \mu_{i}+d_{i} \right)N_{i}$$

*i* ≥ 1, where *d*_100_ = 0.

We set the population size in the model to 1. Death rates were obtained from the WHO database Life Tables for age-specific mortality rates across both genders[2]. The life table data was presented in age groups ranging from one year to five years. We fit the data to obtain one year increments. Other demographic data were obtained from United States Census International Database. For example, the Jordan population in 2015 was assumed to be 8,118,000[3].

The assumption of stationary population structure and size gives the equilibrium relations $\Lambda=(\mu_{0}+d_{0})N_{0}^{*}$and$d_{i-1}N_{i-1}^{*}=(\mu_{i}+d_{i})N_{i}^{*}$. Thus, we need to choose the initial size of age groups as

$$N_{i}\left( 0 \right)=N_{i}^{*}=\Lambda\prod_{j=0}^{i} \frac{d_{j-1}}{d_{j}+\mu_{j}}$$

where *d*_–1_ = 1. To make the size of the population equal to 1, we need to set Λ as

$$\Lambda=(\mu_{0}+d_{0})\left( 1+\sum_{i=1}^{100} \prod_{j=1}^{i} \frac{d_{j-1}}{d_{j}+\mu_{j}} \right)^{-1}$$

# The epidemiologic model

The age-structured epidemiologic model describing the transmission dynamics of hepatitis A virus infections follows an MSEIR (maternal-susceptible-exposed-infectious-recovered) compartmental structure[4] (Figure S1). The model assumes that all infants are born in the *M* class and are protected by maternal antibodies. After the maternal antibodies wanes, the infants move to the susceptible class *S* at rate *ξ*. A fraction of susceptible persons are exposed to infection at an age-specific and time-dependent rate *λ* (per capita force of infection) and enter the exposed compartment. The rate at which persons of a given age class, at a given time are exposed to infection depends on the number of contacts, the fraction of infected contacts, the transmission probability per contact, and the nature of mixing between different age groups. The contact pattern is governed by a conditional probability mixing matrix. Each cell in the mixing matrix represents the probability of a person of a given age class having adequate contact with a person in another age class.

After a latent period, exposed persons can become infectious at rate*δ*. Infection can be either symptomatic according to the age-specific probability *α_j_* or remain asymptomatic with probability 1–*α_j_*. Patients with symptoms are hospitalized (with probability *ε _j_*), can become fulminant (with probability *η_j_*), may require a liver transplantation (with probability *ν _j_*), die from Hepatitis A virus infection (with probability *ς_j_*), or treated as outpatient (with probability 1–*ε _j_*–*η _j_*–*ν _j_*–*ς_j_*). To account for differential mortality, costs, and quality of life during the period immediately following transplantations, we subdivided this health state into two: first year and subsequent years. Infected persons can clear their infection (at rate *σ _j_*) and move to the recovered compartment with lifelong immunity. The recovery rate depends on the infectious class.

Vaccinated persons leave the susceptible compartment (at a given age-specific vaccination rate *φ_j_*) and enter the vaccinated compartment and remain there until they die or their immunity wanes (at rate*γ*). The model assumes that only a fraction, ϕ, of infants receiving the first dose will go on to receive the second dose. Efficacy is assumed to be *ψ*_1_ and *ψ*_2_ for the first-dose only and complete two-dose series, respectively.

The model consists of the following ODEs:

With $i=1, 2, . . ., 100$. The definition of parameters and variables are given in Table S1.

Table S1: Definition of model variables and parameters

| Symbol | Definition |
| --- | --- |
| Variables |  |
| *N_i_* | Number of persons in age group *i* |
| *M_i_* | Number of persons in age group *i* with maternal antibodies |
| *V*_1_*_,i_* | Number of persons in age group *i* receiving 1 dose of the vaccine |
| *V*_2_*_,i_* | Number of persons in age group *i* receiving 2 doses of the vaccine |
| *S_i_* | Number of persons in age group *i* in the susceptible compartment |
| *E_i_* | Number of persons in age group *i* in the exposed compartment |
| *I_i_* | Number of persons in age group *i* in the asymptomatically infected compartment |
| *H_i_* | Number of persons in age group *i* with symptomatic infections hospitalized |
| *F_i_* | Number of persons in age group *i* with fulminant disease |
| *O_i_* | Number of persons in age group *i* with symptomatic infections treated as outpatient |
| *L*_1_*_,i_* | Number of persons in age group *i* who had liver transplant in the last year |
| *L*_2_*_,i_* | Number of persons in age group *i* who had liver transplant more than a year ago |
| *R_i_* | Number of persons in age group *i* the recovered compartment with lifelong immunity |
| *λ_i_* | Force of infection of age group *i* |
| Parameters |  |
| *b*_0_ | Scale parameter for the force of infection |
| *b*_1_ | Shape parameter for the force of infection |
| *b*_2_ | Shape parameter for the force of infection |
| Δ_2_ | Parameter for force of infection at time *t*_2_ |
| Δ_3_ | Parameter for force of infection at time *t*_3_ |
| *t*_1_ | Time for first drop in force of infection begins |
| *t*_2_ | Time for first drop in force of infection ends and second drop begins |
| *t*_3_ | Time for second drop in force of infection ends |
| *ρ* | Underreporting factor |
| *β_ij_* | Contact matrix of mixing between age *i* and *j* |
| *c_i_* | Age-specific activity levels with adequate mixing contact |
| *μ_i_* | Hazard of health for persons in age group *i* |
| *d_i_* | Age-specific transfer rate between age groups |
| Λ | Birth rate |
| 1/*ξ* | Duration of maternal immunity |
| 1/*δ* | Latency period |
| 1/*σ_i,i_* | Age-specific mean duration of infectiousness |
| *σ_o,i_* | Age-specific recovery rate for outpatients |
| *σ_h,i_* | Age-specific recovery rate for hospitalized patients including fulminant |
| *α_i_* | Probability an infection is icteric^§^ |
|  | Scale parameter, *a*_0_ |
|  | Shape parameter, *a*_1_ |
|  | Shape parameter, *a*_2_ |
| *ε_i_* | Age-specific probability of hospitalization of an icteric infection |
| *η_i_* | Age-specific probability of fulminant Hepatitis A from an icteric infection |
| *ν_i_* | Age-specific probability of liver transplant from an icteric infection |
| *ς_i_* | Age-specific probability of death from an icteric infection |
| *θ*_1_ | Rate of death during 1^st^ year of liver transplantation |
| *θ*_2_ | Rate of death from year 2 and beyond after liver transplantation |
| *φ_i_* | Age- and time-specific proportion vaccinated |
| *ψ*_1_ | Vaccine efficacy, 1 dose only |
| *ψ*_2_ | Vaccine efficacy, 2 doses |
| 1/*γ*_1_ | Mean duration of vaccine-derived immunity, 1 dose only |
| 1/*γ*_2_ | Mean duration of vaccine-derived immunity, 2 doses |
| *ϕ* | Vaccine adherence, probability of second dose given first dose |
| §The probability of icteric infection among age group j is given by: as described in[5] | |

Susceptible individuals acquire infection at an age and time dependent rate *λ_i_* known as the force of infection. This rate depends on the age-specific number of adequate contacts *β_ij_*. The force of infection is given by

$$\lambda_{i}=\sum_{j=0}^{100} \beta_{ij}\frac{I_{i}}{N_{j}}.$$

Unlike the U.S. Hepatitis A model, we do not consider a substantial international travel component nor do we include any other exogenous terms to the force of infection. It is reasonable to forgo the travel component in intermediate and high endemicity regions where the local prevalence of disease is expected to be dominate any travel effects as well as the fact that most of the adult population is already immune. Other exogenous effects, such as effects from immigration are not expected to play a significant role.

# Population mixing and transmission rate

The contact matrix *β_ij_* is obtained as follows. We assume separable mixing and use the proportionate mixing form by defining activity levels *c_j_* for each of the 101 age groups. Thus,

$$\beta_{ij}=\frac{c_{i}c_{j}}{\sum_{k=0}^{100} c_{k}N_{k}}.$$

Assuming an equilibrium solution sometime before vaccination, the activity levels *c_j_* are typically found from the equilibrium forces of infection *λ_j_* and the infectious fractions *I_j_* as explained in Hethcote[1]:

$$c_{j}=\frac{\lambda^{*}_{j}\sum_{k=0}^{100} \lambda^{*}_{k}N_{k}}{\sum_{k=0}^{100} \lambda^{*}_{h}I^{*}_{h}/N_{h}}.$$

The epidemiologic data on Hepatitis A in Jordan and other Middle East countries indicates a dynamic system in transition[6–8]. To estimate the forces of infection before vaccination programs were initiated, we used least square methods to fit the model to data on age-specific sero-prevalence and incidence data. Seroprevalence data for 2008 was obtained from a recent study by Hayajneh, et al.[6]. In addition we used seroprevalence data from an older study from approximately 1988[9]. We used total reported incidence data from the Jordan Ministry of Health between 2004 and 2014[10]. In addition we also considered unpublished records from King Abdullah University Hospital covering inpatient cases of hepatitis A between 2003 and 2014. We use this data to represent the proportion of cases that are treated as inpatient cases.

Our approach assumes that the endemic disease reached an equilibrium state and stayed there until the forces of infection began experiencing universal (across all age groups), primarily downward changes during the pre-vaccination period. We assume there are two such changes in force of infection. The first change begins at time t_1_ and ends at t_2_ reducing the force of infection by a factor of Δ_1_. The second change begins at t_2_ and ends at t_3_ reducing the force of infection to the factor Δ_2_[11]. The dates and factors are determined by fitting the model to the sero-prevalence and incidence data. Thus, using a piecewise form, we adjust the force of infection according to$\tilde{\lambda}_{i}(t)=\lambda_{i}(t)\times f(t),$ where

$$\begin{matrix} \mathbf{f}\left( \boldsymbol{t} \right)\mathbf{=} & \left\{ \begin{matrix} \mathbf{1} & \boldsymbol{t}\boldsymbol{\leq}\boldsymbol{t}_{\mathbf{1}} \\ \mathbf{1+}\left( \boldsymbol{t}\mathbf{-}\boldsymbol{t}_{\mathbf{1}} \right)\left( \boldsymbol{\Delta}_{\mathbf{1}}\mathbf{-1} \right)\boldsymbol{/(}\boldsymbol{t}_{\boldsymbol{2}}\boldsymbol{-}\boldsymbol{t}_{\boldsymbol{1}}\boldsymbol{)} & \boldsymbol{t}_{\mathbf{1}}\mathbf{<}\boldsymbol{t}\boldsymbol{\leq}\boldsymbol{t}_{\mathbf{2}} \\ \boldsymbol{\Delta}_{\mathbf{1}}\mathbf{+}\left( \boldsymbol{t}\mathbf{-}\boldsymbol{t}_{\mathbf{2}} \right)\left( \boldsymbol{\Delta}_{\mathbf{2}}\mathbf{-}\boldsymbol{\Delta}_{\mathbf{1}} \right)\boldsymbol{(}\boldsymbol{t}_{\boldsymbol{3}}\boldsymbol{-}\boldsymbol{t}_{\boldsymbol{2}}\boldsymbol{)} & \boldsymbol{t}_{\mathbf{2}}\mathbf{<}\boldsymbol{t}\boldsymbol{\leq}\boldsymbol{t}_{\boldsymbol{3}} \\ \boldsymbol{\Delta}_{\mathbf{2}} & \boldsymbol{t}\mathbf{>}\mathbf{t}_{\boldsymbol{3}} \end{matrix} \right. \end{matrix}$$

This function accounts for overall (non-age-specific) changes in the level of HAV endemicity in Jordan. In the U.S., these changes occurred primarily in the early part of the 20^th^ century and the resulting overall endemicity in the U.S. was extremely low, essentially in equilibrium by the time a vaccine was available in the 1990s. In Jordan, these changes have occurred in the more recent past and may be still ongoing. Key drivers are likely to be socioeconomic development and improved sanitation and water quality as well as broadening availability of each


[8–10]. These improvements began in the late 1970s and continued into the 2000s when more substantial and widely available improvement occurred[12,13]. More recently, however, however, the decrease in force of infection appears to have stopped which may be due to a number of factors including the Syrian refugee situation and water shortages potentially straining sanitation and supply systems[14–16]. The expectation is that t_1_, t_2_ , and t_3_ will be dates in the 1980s, 1990s, and late 2000s respectively.

The equilibrium forces of infection $\lambda^{*}_{j}$ are assumed to take the gamma form[17]

$$\lambda^{*}_{j}=b_{0}j^{b_{1}}e^{(-j/b_{2})}.$$

Because a high proportion of Hepatitis A virus infections are anicteric (i.e. are not recognized), we adjusted the model predicted disease incidence by the age-specific probability of developing jaundice *α_j_*[5]:

$$\alpha_{j}=0.852\left[1-Exp(-0.01244j^{1.903})\right].$$

To account for underreporting when fitting the incidence data we include an underreporting factor*ρ (5)*. Additionally, we assume that all underreported cases are ultimately treated.

# Model fitting procedure

To simplify the fitting procedure, we reformulated the model by excluding all symptomatic infections (those in classes *H*, *F*, *O*, *L*_1_, *L*_2_) and focusing only on variables that are relevant for transmission or immunity (those in classes *M*, *S*, *E*, *I*, *R*). We also define a new variable *P_j_* for all persons who are currently exposed, currently infected, or previously infected: *P_j_* = *M_j_* + *E_j_* + *I_j_* + *R_j_*. Because we assumed a stationary population we have *S_j_* = *N_j_* – *P_j_* for all *j* = 0, 1, 2, …, 100. The model without vaccination is

$$\begin{matrix} dM_{0}/dt= \Lambda-(\xi+\mu_{0}+d_{0})M_{0}, \\ dM_{j}/dt= d_{j-1}M_{j-1}-(\xi+\mu_{j}+d_{j})M_{j}, \\ dE_{0}/dt= f\lambda_{0}(N_{0}-P_{0})-(\delta+\mu_{0}+d_{0})E_{0}, \\ dE_{j}/dt= d_{j-1}E_{j-1}+f\lambda_{j}(N_{j}-P_{j})-(\delta+\mu_{j}+d_{j})E_{j}, \\ dI_{0}/dt= (1-\alpha_{0})\delta E_{0}-(\sigma_{i,0}+\mu_{0}+d_{0})I_{0}, \\ dI_{j}/dt= d_{j-1}I_{j-1}+(1-\alpha_{j})\delta E_{j}-(\sigma_{i,j}+\mu_{j}+d_{j})I_{j}, \\ dP_{0}/dt= \Lambda+f\lambda_{0}(N_{0}-P_{0})-(\mu_{0}+d_{0})P_{0}-\xi M_{0}, \\ dP_{j}/dt= d_{j-1}P_{j-1}+f\lambda_{j}(N_{j}-P_{j})-(\mu_{j}+d_{j})P_{j}-\xi M_{j}, \\ dN_{0}/dt= \Lambda-(\mu_{0}+d_{0})N_{0}, \\ dN_{j}/dt= d_{j-1}N_{j-1}-(\mu_{j}+d_{j})N_{j}. \end{matrix}$$

The initial conditions for this model are given by the equilibrium values:

$$\begin{matrix} M_{0}(0)= M^{*}_{0}= \Lambda/(\xi+\mu_{0}+d_{0}), \\ M_{j}(0)= M^{*}_{j}=d_{j-1}M^{*}_{j-1}/(\xi+\mu_{j}+d_{j}), \\ P_{0}(0)= P^{*}_{0}=(\lambda^{*}_{0}N^{*}_{0}+\Lambda-\xi M^{*}_{0})/(\lambda^{*}_{0}+\mu_{0}+d_{0}), \\ P_{j}(0)= P^{*}_{j}= [d_{j-1}P^{*}_{j-1}+\lambda^{*}_{j}N_{j}-\xi M^{*}_{j}]/(\lambda^{*}_{j}+\mu_{j}+d_{j})P_{j}, \\ E_{0}(0)= E^{*}_{0}=\lambda^{*}_{0}(N^{*}_{0}-P^{*}_{0})/(\delta+\mu_{0}+d_{0}), \\ E_{j}(0)= E^{*}_{j}=[d_{j-1}E^{*}_{j-1}+\lambda^{*}_{j}(N_{j}-P^{*}_{j})]/(\delta+\mu_{j}+d_{j}), \\ I_{0}(0)= I^{*}_{0}=(1-\alpha_{0})\delta E^{*}_{0}/(\sigma_{i,0}+\mu_{0}+d_{0}), \\ I_{j}(0)= I^{*}_{j}=[d_{j-1}I^{*}_{j-1}+(1-\alpha_{j})\delta E^{*}_{j}]/(\sigma_{i,j}+\mu_{j}+d_{j}), \\ N_{0}(0)= N^{*}_{0}= \Lambda/(\mu_{0}+d_{0}), \\ N_{j}(0)= N^{*}_{j}= d_{j-1}N^{*}_{j-1}/(\mu_{j}+d_{j}). \end{matrix}$$

The equilibrium forces of infection *λ_j_* is given by

$$\lambda^{*}_{j}=b_{0}j^{b_{1}}e^{(-j/b_{2})}.$$

We fixed all other parameters and used the nine parameters, *b*_0_, *b*_1_, *b*_2_, Δ_1_, Δ_2_, *t*_1_, *t*_2_, t_3_ and *ρ* for fitting the model. As a function of these nine parameters, the model-predicted reported incidence in all age groups at time *t* is given by:

$$\hat{Y}(t)=\text{(}1/\rho)\sum_{j=0}^{100} \alpha_{j}\delta E_{j}(t).$$

The model-predicted seroprevalence in age group *j* at time *t* is given by:

$$\hat{Z}_{j}(t)=P_{j}(t)/N_{j}(t)$$

Our calibration approach will be to minimize a least square function consisting of three components. The first is the least square deviation in age-specific seroprevalence predicted by the model for 2008 and from the seroprevalence from Hayajneh, et al.[6] for Jordan in 2008 and represented as $Z_{i}\left( 2008 \right)$. The second component is the least square deviation between seroprevalence predicted by the model for 1988 and from the seroprevalence data from Toukan, et al.[9] for Jordan in 1988 and represented as $Z_{i}\left( 1988 \right)$. The final component is the least square deviation between model predicted incidence from 2004 through 2014 from the Jordanian Ministry of Health[10] represented by $Y(t)$. We standardize the components by dividing each element by the standard deviation of the measured data. The resulting function to be minimized is

$$RSS=\frac{\sum_{t=2004}^{2014} \left[ Y\left( t \right)-\hat{Y}(t) \right]^{2}}{\text{var}[\left\{ Y_{j}(t) \right\}_{t=\{2004,\ldots,2014\}}]}+\frac{\sum_{j=\{0,1,2,5,10,15,20\}} \left[ Z_{j}(2008)-\hat{Z}_{j}(2008) \right]^{2}}{\text{var}[\left\{ Z_{j}(2008) \right\}_{j=\{0,1,2,5,10,15,20\}}]}+\frac{\sum_{j=\{0,1,2,3,4,5,10,20,40\}} \left[ Z_{j}(1988)-\hat{Z}_{j}(1988) \right]^{2}}{\text{var}[\left\{ Z_{j}(1988) \right\}_{j=\{0,1,2,3,4,5,10,20,40\}}]}$$

We use Mathematica 10.2 for all simulations and analysis including model fitting. The build-in function, NMinimize was utilized to minimize RSS with respect to the parameters *b*_0_, *b*_1_, *b*_2_, Δ_1_, Δ_2_,, *t*_1_, *t*_2_, t_3_, and *ρ.* The results are presented in Table S2. Plots of data and fitted curves are in the Calibration Results appendix.

### Estimating the Proportion of Hospitalization Outcomes

In addition, we used unpublished HAV hospitalization data from King Abdullah University Hospital (KAUH) in Irbid, Jordan to estimate the proportion of HAV cases that required inpatient care (see Analysis of KAUH data in Appendix for details)

Further we assumed that all unreported cases were treated and therefore required health-care resources. We used the KAUH data and the calculated underreporting factor to estimate the proportion of the treated cases that were in-patient and assumed the rest were treated as outpatients. The assumption that was made in the U.S. HAV model was that all unreported cases were treated and that all reported cases represented inpatient treatment, the rest being treated as outpatient[11]. We use the following rationale:

In general, we can assume that the number of cases of any infections disease can be split into a proportions representing those reported to a surveillance system (
R), those that are un-reported (UR), and those that are un-assessed (UA)[18]. Further the underestimation factor is UE = UR + UA. The factor UA general represents both symptomatic and asymptomatic cases that do not get treated. In the case of the HAV model, we have a factor to separate out the asymptomatic cases so we can then consider the factor UA’ as representing all symptomatic, un-assessed cases. The assumption that all underestimated cases are assessed and therefore treated is UA’ = 0, and UE = UR. Using this framework we have the proportion of under-reported cases which is

UE = UR = Reported Cases / All symptomatic cases

To determine the number of cases treated as inpatient we use the ratio of

[KAUH(t)/MOH(t)]_avg_ = Hospitalized Cases/Reported Cases

We used the years 2005 through 2013 to determine this ratio. We can then use this ratio to modify the calculated under reporting factor, ρ, to be used as the hospitalization factor

$\rho^{'}=\rho\left[ \frac{KAUH(t)}{MOH(t)} \right]_{\text{avg}}=\frac{\text{All Cases}}{Reported Cases} \left[ \frac{KAUH(t)}{MOH(t)} \right]_{\text{avg}}=24.9451*\frac{1}{2.1209}= 11.7616=\frac{\text{All Cases}}{\text{Hospitalized Case }}$

We use this factor to estimate the proportion of symptomatic (icteric) incident cases that lead to hospitalization using

$$I_{hospitalized}=H+L1+F+ I_{death}= \frac{I_{symptomatic}}{\rho^{'}}\sim0.085 I_{symptomatic}$$

The rest of the symptomatic cases are considered outpatient

$$I_{outpatient}=O= \frac{(\rho^{'}-1)}{\rho^{'}}I_{symptomatic}\sim0.915 I_{symptomatic}$$

The hospitalization probability is further divided into probability of icteric infection resulting in hospitalization, fulminant infection, liver transplant, death using conditional probabilities from Rein, et al.[19], resulting in the values presented in Table 1 of the main manuscript.

Table S2: Parameter values for force of infection functions and under reporting factor

| **Parameter** | **Symbol** | **Value** |
| --- | --- | --- |
| Scale parameter for the force of infection | *b*_0_ | 0.901466 |
| Shape parameter for the force of infection | *b*_1_ | 0.00207163 |
| Shape parameter for the force of infection | *b*_2_ | 12.14448 |
| Magnitude of change in force of infection between t_1_ and *t*_2_ | Δ_1_ | 0.43213 |
| Magnitude of change in force of infection between t_2_ and t_3_ | Δ_2_ | 0.321572 |
| Time first change in magnitude of force of infection begins infection *t*_1_ | *t*_1_ | 1983.8 |
| Time second change in magnitude of force of infection begins | *t*_2_ | 1998.9 |
| Time second change in magnitude of force of infection ends | *t_3_* | 2009 |
| Underreporting factor | *ρ* | 24.9541 |
| Hospitalization factor | *ρ’* | 11.7616 |

# Choice of distributions for probabilistic sensitivity analysis (PSA)

In this section we briefly discuss the rationale for choosing distributions and the methods for estimating their parameters.

## Gamma Distributions

Gamma distribution is appropriate for non-negative, skewed data sets. The parameters of the gamma distribution were obtained using the method of moments. Given the mean (*m*), low (*l*), and high (*h*) values of the parameter as shown in Table 1 in the manuscript, we estimate the standard error (*se*) as

$$se= \frac{h-l}{2(1.96)}$$

The parameters of the Gamma distribution are given by

$$\begin{matrix} \alpha& = & \frac{m^{2}}{{se}^{2}}, \\ \beta& = & \frac{{se}^{2}}{m} \end{matrix}$$

### Costs

Because costs are constrained to be non-negative and are highly skewed, the gamma distribution was chosen for representing uncertainty in cost parameters[20].

### Duration of protection

The parameter representing median duration of protection is constrained between 0 and infinity. This suggests gamma as a reasonable distribution for representing uncertainty regarding duration of protection.

## Beta Distributions

Beta distributions are appropriate for values constrained to be between zero and one. The parameters for the beta distribution were obtained as

$$\begin{matrix} \alpha& = & \frac{m(m-m^{2}-{se}^{2})}{{se}^{2}}, \\ \beta& = & \frac{{(1-m)(m-m^{2}-se}^{2})}{{se}^{2}} \end{matrix}$$

### Health Related Quality of life

Because utilities or health-related quality of life (QoL) weights in this analysis are assumed to be between 0 and 1, we used a beta distribution to represent uncertainty in QoL parameters. Base case values were used as mean of beta distribution and standard error was estimated using equation 1 above. The upper and lower confidence limits for Hepatitis A QALY were 0.6076 and 0.6819 respectively[21]. For QALY of liver transplant patients, we used 0.84 and 0.63 as upper and lower confidence limits[22].

### Distribution of vaccine uptake and adherence rates

In the model, vaccine uptake (first dose) and adherence (second dose) were represented by a proportion of persons moving to older age groups. This proportion can take a value between 0 and 1. Thus, the beta distribution is a reasonable choice for representing uncertainty in vaccine uptake parameters. We assumed a lower limit of 0.8, upper limit of 0.97, and a mean of 0.9. For the proportion receiving second dose given the first dose we assumed a lower limit of 0.7, upper limit of 0.97, and a mean of 0.95.

## Dirichlet distributions

The variability in health outcomes from Hepatitis A was modeled using a Dirichlet distribution. We used the probabilities of hospitalization, fulminant disease, death from liver transplant, and liver transplant from base case to derive numbers for Dirichlet distribution. We used the age-specific data derived from conditional probabilities in Rein, et al. [19]. All inpatient probabilities were modified by dividing by the hospitalization parameter, ρ’, discussed above. These values were used as medians to determine the parameters for the distributions. The resulting Dirichlet distributions are a (Table 3).

Table 3: parameters for the Dirichlet distributions.

|  |  | Numbers for Dirichlet distribution | | | | |
| --- | --- | --- | --- | --- | --- | --- |
|  |  | **Outpatient** | **Hospitalized** | **Fulminant** | **Death** | **Liver transplant** |
| **D1** | **≤4** | 97.0207 | 0.385265 | 0.00456518 | 0.031484 | 0.00590325 |
| **D2** | **5–14** | 349.97 | 5.13289 | 0.00218903 | 0.0150967 | 0.00283064 |
| **D3** | **15–39** | 458.787 | 8.95484 | 0.0392421 | 0.270635 | 0.0507441 |
| **D4** | **40–59** | 377.566 | 6.07215 | 0.261759 | 1.80523 | 0.338481 |
| **D5** | **60–95** | 161.413 | 2.82536 | 0.757196 | 1.13014 | 0.0282536 |

## Distributions for vaccine properties

The properties of the vaccine include degree of protection against infection, and degree of protection against disease given a breakthrough infection. The degree of protection is 1 minus residual susceptibility (i.e., relative risk of infection among vaccine and placebo recipients). Because the relative risk ranges from zero to infinity, the appropriate distribution for residual susceptibility parameters is a lognormal distribution[20]. Because the mean relative risk is zero, we used a derived distribution to represent the uncertainty in degree of protection against infection.

# Sample size

We used Monte Carlo Latin hypercube sampling (LHS). LHS is recommended for use with computationally demanding models such as this because its efficient stratification properties allow for the extraction of a large amount of uncertainty and sensitivity information with a relatively small sample size[23]. In LHS, the random parameter distributions are divided into *N* equal probability intervals. Each interval for each parameter is sampled (without replacement) exactly once, so that the entire range for each parameter is explored. The choice for *N* should be at least *k*+1, where *k* is the number of parameters varied, but usually *N* is selected to be much larger than *k*+1 to ensure accuracy. Empirically the following inequality should be satisfied: N >4*k/3[23]. In our analysis, we chose 1000 random samples for use as inputs in the simulations. Given the number of parameters (48 parameters) included in the analysis, the empirical inequality is clearly satisfied.

# References

[1] Hethcote HW. An age-structured model for pertussis transmission. Math Biosci 1997;145:89–136.

[2] World Health Organization. Global Health Observatory: Life Tables by Country Jordan 2015. http://apps.who.int/gho/data/?theme=main&vid=60830 (accessed November 11, 2015).

[3] United States Census Bureau. International Database: Demographics: Jordan 2015. http://www.census.gov/population/international/data/idb/region.php?N= Results &T=13&A=separate&RT=0&Y=2010,2011,2012,2013,2014,2015&R=-1&C=JO (accessed September 24, 2015).

[4] Hethcote HW. The Mathematics of Infectious Diseases. SIAM Rev 2000;42:599–653.

[5] Armstrong GL, Bell BP. Hepatitis A Virus infections in the United States: Model-Based Estimates and Implications for Childhood Immunization. Pediatrics 2002;109.

[6] Hayajneh WA, Balbeesi A, Faouri S. Hepatitis A virus age-specific sero-prevalence and risk factors among Jordanian children. J Med Virol 2015;87:569–74.

[7] Itani T, Jacobsen KH, Nguyen T, Wiktor SZ. A new method for imputing country-level estimates of hepatitis A virus endemicity levels in the Eastern Mediterranean region. Vaccine 2014;32:6067–74.

[8] Melhem NM, Talhouk R, Rachidi H, Ramia S. Hepatitis A virus in the Middle East and North Africa region: a new challenge. J Viral Hepat 2014;21:605–15.

[9] Toukan AU, Sharaiha ZK, Abu-el-Rob OA. The seroepidemiology of hepatitis A virus infection in Jordan. Trop Gastroenterol 1988;9:76–9.

[10] Directorate of Vital information Studies Preparation. Ministry of Health Annual Statistical Book 2014. Amman, Jordan: Jordan Ministry of Health; 2015.

[11] Dhankhar P, Nwankwo C, Pillsbury M, Lauschke A, Goveia MG, Acosta CJ, et al. Public Health Impact and Cost-Effectiveness of Hepatitis A Vaccination in the United States: A Disease Transmission Dynamic Modeling Approach. Value Heal 2015;18:358–67.

[12] Jiries A. Water resources in Jordan. Adv. Water Supply Wastewater Treat. A Road to Safer Soc. Environ., Springer; 2011, p. 193–9.

[13] WHO & UNICEF. Progress on Sanitation and Drinking Water: 2015 Update and MDG Assessment. UNICEF; 2015.

[14] Slaih AA. Impact of Syrian Refugees on Jordan’s health sector 2013.

[15] UN Refugee Agency. Syrian Regional Refugee Response: Inter-agency Information Sharing Portal 2015.

[16] Carrion D. Syrian Refugees in Jordan: Confronting Difficult Truths. London: 2015.

[17] Whitaker H. J. Farrington CP. Estimation of infections disease parameters from serological survey data: the impact of regular epidemics. Stat Med 2004;23:2429–43.

[18] Gibbons CL, Mangen M-JJ, Plass D, Havelaar AH, Brooke RJ, Kramarz P, et al. Measuring underreporting and under-ascertainment in infectious disease datasets: a comparison of methods. BMC Public Health 2014;14:147.

[19] Rein DB, Hicks KA, Wirth KE, Billah K, Finelli L, Fiore AE, et al. Cost-effectiveness of routine childhood vaccination for hepatitis A in the United States. Pediatrics 2007;119:e12–21.

[20] Briggs A, Sculpher M, Claxton K. Decision modelling for health economic evaluation. Oxford university press; 2006.

[21] Luyten J, Marais C, Hens N, De Schrijver K, Beutels P. Imputing QALYs from single time point health state descriptions on the EQ-5D and the SF-6D: a comparison of methods for hepatitis A patients. Value Heal 2011;14:282–90.

[22] Chong CAKY, Gulamhussein A, Heathcote EJ, Lilly L, Sherman M, Naglie G, et al. Health-state utilities and quality of life in hepatitis C patients. Am J Gastroenterol 2003;98:630–8.

[23] Blower SM, Dowlatabadi H. Sensitivity and uncertainty analysis of complex models of disease transmission: an HIV model, as an example. Int Stat Rev Int Stat 1994:229–43.

[24] Battikhi MN, Battikhi EG. The seroepidemiology of hepatitis A virus in Amman, Jordan. New Microbiol 2004;27:215–20.

# Appendix

## Model Structure

φϕ

(1−ψ_1_)λ

(1−ψ_2_)λ

γ_2_

One Dose Vaccinated

*(V1*)

Susceptible

(*S*)

Exposed

(*E*)

Two Doses Vaccinated

*(V2*)

Maternal

(*M*)

Asymptomatic

(*I*)

Hospitalized

(*H*)

Liver Transplant (*L*_1_)

Fulminant

(*F*)

Outpatient

(*O*)

Recovered

(*R*)

Post Liver Transplant

(*L*_2_)

Death from liver disease

(*D*)

ξ

φ(1-ϕ)

γ_1_

λ

(1−α)δ

εαδ

ηαδ

ναδ

(1−ε−η−ν−ζ)αδ

ζαδ

σ_i_

σ_h_

σ_o_

σ_f_

θ_1_

θ_2_

Figure S1: Flow diagram of hepatitis A virus transmission and vaccination model: newborns enter age group a = 0, and are assumed to be protected by maternal antibodies (*M*), the protection is lost over time so the children become susceptible (*S*). Upon infection, a person moves to the exposed compartment (*E*) becomes infectious after a latent period. The model distinguishes between several categories of infection and disease: Asymptomatic (*I*), Symptomatic infections are treated as outpatient (*O*), hospitalized (*H*), with fulminant disease (*F*), requiring a liver transplant (*L*), or die from Hepatitis A virus infection (*D*). Infected persons can clear their infection and move to the recovered compartment (*R*) with lifelong immunity. Susceptible individuals may be vaccinated and move to the Vaccinated (V) compartment. Vaccinated individuals may more back to the susceptible (S) compartment due to waning vaccine or to the exposed compartment due to breakthrough infections. The model also applied age-specific all-cause mortality (not shown) to all persons in all epidemiologic classes.

## Calibration Results

The model was calibrated against the three sets of results discussed in the text. Figures **Error! Reference source not found.Error! Reference source not found.** were produced using the calibration data and the no-vaccine model using the calibration parameters from the Table S2.


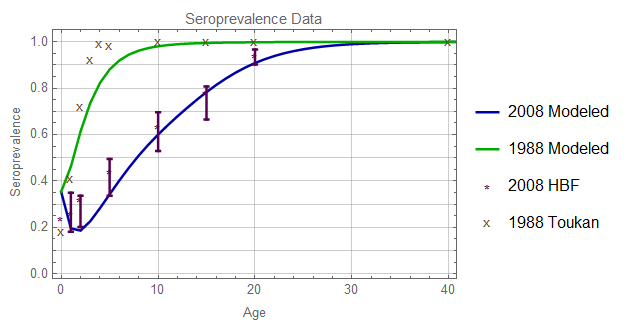


Figure 2: Seroprevalence data from Hayajneh, et al. (2008 HBF) and Toukan, et al. (1988 Toukan). The error bars on 2008 HBF are derived from the odd ratio confidence intervals presented in Hayjneh, et al. The data for 1988 Toukan contained no confidence interval information and were only available by extraction from a plot in the Toukan, et al[9].


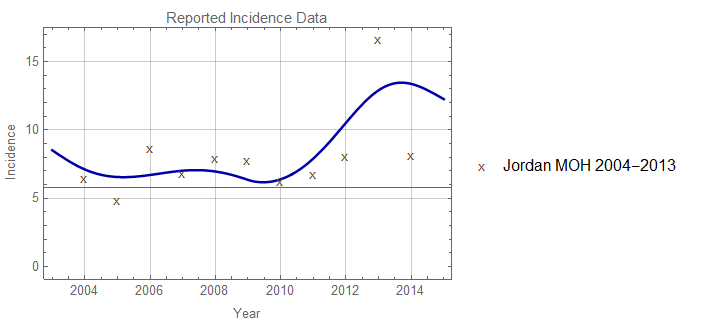


Figure 3: Incidence data from Jordan Ministry of Health 2004 and 2014

##

## Model Validation against epidemiologic data

We compare the calibrated model incidence predictions to data available from Battikhi & Battikhi from a study of HAV incidence in Amman Jordan[24]. Since this data from a specific city we should not expect detailed correspondence with the country wide model predictions. The modeled results are a reasonably good fit to the average incidence over this time period.


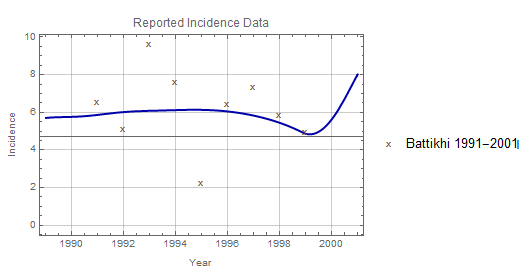


Figure 4: Model reported incidence results compared to results from Amman Jordan between 1991 and 1999.

## Analysis of KAUH data

Raw unpublished admissions data from King Abdullah University Hospital (KAUH) in Irbid, Jordan was available to derive the incidence rates. We first identified all patients in each year represented in the data that tested positive for Ig-M. We then determined the proportion of the total entire Jordanian population that is served by KAUH by considering the number of hospital beds in KAUH relative to the total number of hospital beds in all of Jordan. We also considered the total overall admissions in KAUH relative to the total overall admissions to all hospitals in Jordan. We then used the average of these two ratios to estimate the proportion of the Jordan population represented in the KAUH data. The analysis lead to a ratio of approximately 4%. We combined this with the total population of Jordan from the US Census Bureau International Database


[16] to calculate the number of HAV patients per 100,000.

The results are presented in Table S1. The table clearly shows a substantial increase in incidence from 2003 through 2013 as well as some oscillation in incidence. The oscillations are an expected result for the non-equilibrium state affected by the overall changes in endemicity driven by socioeconomic improvements in the recent past. If we assume that KAUH is representative in Jordan as a whole and that there are no significant confounding factors such as a national HAV awareness campaign, then there must be a universal (across all age groups) increase in force of infection. This is represented in the Eq. 1. There is some expectation of an overall increase in incidence due to the influx of Syrian refugees over the past six years. It is also possible that KAUH may see a larger effect from the refugee situation since it is in the northern part of the country where there tend to be more Syrian refugees


[15].

Table S4: incidence of HAV for Jordan derived from KAUH admissions data. The calculations assume that KAUH covers 4% of the Jordan population as described in the text.

| **Year** | **Patients with HAV at HAUK** | **Jordan Population** | **HAUK Portion of Population** | **# Per 100000** |
| --- | --- | --- | --- | --- |
| 2003 | 4 | 5013775 | 200551 | 1.99 |
| 2004 | 4 | 5128263 | 205131 | 1.95 |
| 2005 | 21 | 5245404 | 209816 | 10.01 |
| 2006 | 25 | 5617215 | 224689 | 11.13 |
| 2007 | 44 | 5997193 | 239888 | 18.34 |
| 2008 | 30 | 6132625 | 245305 | 12.23 |
| 2009 | 42 | 6269285 | 250771 | 16.75 |
| 2010 | 38 | 6407085 | 256283 | 14.83 |
| 2011 | 37 | 6508271 | 260331 | 14.21 |
| 2012 | 58 | 6508887 | 260355 | 22.28 |
| 2013 | 87 | 6482081 | 259283 | 33.55 |
| 2014 | 40 | 6528061 | 261122 | 15.32 |

Data Tables

Table 5: One way sensitivity analysis results. The “Ranges” columns show the absolute value of the range from high to low for each of the three result values. Red indicates the largest magnitude change and white the smallest.

|  | **Low Values** | | | | **High Values** | | | | | **Ranges** | | | |
| --- | --- | --- | --- | --- | --- | --- | --- | --- | --- | --- | --- | --- | --- |
| **Parameter** | **Indirect Costs** | **Total Costs** | **QALY** | **Years to Cost Savings** | | **Indirect Costs** | **Total Costs** | **QALY** | **Years to Cost Savings** | | **Years to Cost Saving** | **Costs** | **QALYs** |
| **Outpatient cost** | -1.72803 | -0.158283 | 0.00462 | 45.5259 | | -1.72803 | -8.09278 | 0.00462 | 3.24884 | | 42.28 | 7.934497 | 0 |
| **Vaccine Cost** | -1.72803 | -6.3408 | 0.00462 | 3.82902 | | -1.72803 | -3.06186 | 0.00462 | 13.8851 | | 10.06 | 3.27894 | 0 |
| **Hospitalization costs** | -1.72803 | -4.85375 | 0.00462 | 6.0563 | | -1.72803 | -5.97651 | 0.00462 | 4.62459 | | 1.43 | 1.12276 | 0 |
| **Vaccine adherence, second dose give first dose (%)** | -1.72801 | -6.08206 | 0.00462 | 4.14974 | | -1.72803 | -5.24782 | 0.00462 | 5.43783 | | 1.29 | 0.83424 | 1E-07 |
| **Work loss days, outpatient** | -0.821896 | -4.34169 | 0.00462 | 6.35086 | | -1.99454 | -5.51433 | 0.00462 | 5.2375 | | 1.11 | 1.17264 | 0 |
| **median monthly earnings 25+** | -1.35629 | -4.87608 | 0.00462 | 5.67741 | | -2.66064 | -6.18044 | 0.00462 | 4.95335 | | 0.72 | 1.30436 | 0 |
| **labor force participation 16-19** | -1.68774 | -5.20754 | 0.00462 | 5.49656 | | -2.19364 | -5.71343 | 0.00462 | 4.85835 | | 0.64 | 0.50589 | 0 |
| **median monthly earnings <25** | -1.34382 | -4.86362 | 0.00462 | 5.94076 | | -1.82952 | -5.34931 | 0.00462 | 5.31661 | | 0.62 | 0.48569 | 0 |
| **labor force participation 20-24** | -1.6881 | -5.20789 | 0.00462 | 5.48595 | | -2.27283 | -5.79262 | 0.00462 | 4.87917 | | 0.61 | 0.58473 | 0 |
| **Discount Rate** | -3.70753 | -12.3694 | 0.009208 | 5.21143 | | -1.13899 | -3.20718 | 0.003214 | 5.60776 | | 0.40 | 9.16222 | 0.005994 |
| **Vaccine uptake, first dose (%)** | -1.72543 | -5.21598 | 0.004605 | 5.7118 | | -1.72832 | -5.25139 | 0.004622 | 5.40718 | | 0.30 | 0.03541 | 1.69E-05 |
| **Fulminant costs** | -1.72803 | -5.14316 | 0.00462 | 5.54953 | | -1.72803 | -5.35222 | 0.00462 | 5.33221 | | 0.22 | 0.20906 | 0 |
| **Vaccine efficacy, 2 dose only (%)** | -1.72608 | -5.22389 | 0.004608 | 5.64356 | | -1.72803 | -5.24782 | 0.00462 | 5.43783 | | 0.21 | 0.02393 | 1.14E-05 |
| **Liver transplant cost 1st year** | -1.72803 | -5.1544 | 0.00462 | 5.51757 | | -1.72803 | -5.34125 | 0.00462 | 5.3618 | | 0.16 | 0.18685 | 0 |
| **Public health costs** | -1.72803 | -5.24782 | 0.00462 | 5.43783 | | -1.84228 | -5.36208 | 0.00462 | 5.28344 | | 0.15 | 0.11426 | 0 |
| **Probability of death 1st year liver transplant** | -1.72803 | -5.37315 | 0.004618 | 5.41683 | | -1.72803 | -4.64695 | 0.004629 | 5.55715 | | 0.14 | 0.7262 | 1.04E-05 |
| **labor force participation 25-34** | -1.62664 | -5.14643 | 0.00462 | 5.51242 | | -1.81494 | -5.33473 | 0.00462 | 5.37636 | | 0.14 | 0.1883 | 0 |
| **Liver transplant cost 2nd+ year** | -1.72803 | -4.85642 | 0.00462 | 5.49508 | | -1.72803 | -5.63923 | 0.00462 | 5.38381 | | 0.11 | 0.78281 | 0 |
| **Probability of death after 1st year liver transplant** | -1.72803 | -5.66393 | 0.004614 | 5.43069 | | -1.72803 | -4.47844 | 0.004632 | 5.46091 | | 0.03 | 1.18549 | 1.79E-05 |
| **Work loss days, inpatient** | -1.6803 | -5.2001 | 0.00462 | 5.47656 | | -1.71116 | -5.23095 | 0.00462 | 5.45142 | | 0.03 | 0.03085 | 0 |
| **labor force participation 35-44** | -1.69652 | -5.21631 | 0.00462 | 5.44426 | | -1.7698 | -5.28959 | 0.00462 | 5.42934 | | 0.01 | 0.07328 | 0 |
| **Vaccine efficacy, 1 dose only (%)** | -1.72794 | -5.2467 | 0.004619 | 5.44743 | | -1.72803 | -5.24782 | 0.00462 | 5.43783 | | 0.01 | 0.00112 | 5.4E-07 |
| **Median duration of vaccine-derived immunity, 2 dose** | -1.72802 | -5.24777 | 0.00462 | 5.43831 | | -1.72803 | -5.24789 | 0.00462 | 5.43726 | | 0.00 | 0.00012 | 6E-08 |
| **labor force participation 45-55** | -1.71837 | -5.23816 | 0.00462 | 5.43826 | | -1.73753 | -5.25732 | 0.00462 | 5.4374 | | 0.00 | 0.01916 | 0 |
| **Work loss days, fulminant** | 1.74979 | -5.24569 | 0.00462 | 5.43904 | | -1.72727 | -5.24707 | 0.00462 | 5.43826 | | 0.00 | 0.00138 | 0 |
| **labor force participation 55-64** | -1.72416 | -5.24395 | 0.00462 | 5.43791 | | -1.73197 | -5.25177 | 0.00462 | 5.43774 | | 0.00 | 0.00782 | 0 |
| **Work loss days, liver 1st yr 18 - 40** | -1.72795 | -5.24774 | 0.00462 | 5.43789 | | -1.72812 | -5.24792 | 0.00462 | 5.43775 | | 0.00 | 0.00018 | 0 |
| **Median duration of vaccine-derived immunity, 1 dose only** | -1.72803 | -5.24782 | 0.00462 | 5.43786 | | -1.72803 | -5.24783 | 0.00462 | 5.43778 | | 0.00 | 1E-05 | 0 |
| **labor force participation 65+** | -1.72793 | -5.24772 | 0.00462 | 5.43783 | | -1.72816 | -5.24796 | 0.00462 | 5.43782 | | 0.00 | 0.00024 | 0 |
| **Work loss days, liver 1st yr 0-17** | -1.72803 | -5.24782 | 0.00462 | 5.43783 | | -1.72803 | -5.24782 | 0.00462 | 5.43782 | | 0.00 | 0 | 0 |
| **Work loss days, liver 1st yr 41 -55** | -1.72804 | -5.24783 | 0.00462 | 5.43782 | | -1.72807 | -5.24787 | 0.00462 | 5.43782 | | 0.00 | 4E-05 | 0 |
| **Duration of outpatient icteric infection (d)** | -1.72803 | -5.24782 | 0.003247 | 5.43783 | | -1.72803 | -5.24782 | 0.005061 | 5.43783 | | 0.00 | 0 | 0.001814 |
| **Population norms, 0-29** | -1.72803 | -5.24782 | 0.004586 | 5.43783 | | -1.72803 | -5.24782 | 0.004653 | 5.43783 | | 0.00 | 0 | 6.69E-05 |
| **Duration of inpatient icteric infection (d)** | -1.72803 | -5.247820 | 0.004568 | 5.43783 | | -1.72803 | -5.247820 | 0.004633 | 5.43783 | | 0.00 | 0 | 6.42E-05 |
| **Population norms, 30-39** | -1.72803 | -5.24782 | 0.004619 | 5.43783 | | -1.72803 | -5.24782 | 0.004621 | 5.43783 | | 0.00 | 0 | 2.36E-06 |
| **Population norms, 40-49** | -1.72803 | -5.24782 | 0.004619 | 5.43783 | | -1.72803 | -5.24782 | 0.00462 | 5.43783 | | 0.00 | 0 | 8.4E-07 |
| **Population norms, 50-59** | -1.72803 | -5.24782 | 0.00462 | 5.43783 | | -1.72803 | -5.24782 | 0.00462 | 5.43783 | | 0.00 | 0 | 1.1E-07 |
| **Population norms, 60-69** | -1.72803 | -5.24782 | 0.00462 | 5.43783 | | -1.72803 | -5.24782 | 0.00462 | 5.43783 | | 0.00 | 0 | 1E-08 |
| **Work loss days, liver 1st yr 63+** | -1.72803 | -5.24782 | 0.00462 | 5.43783 | | -1.72803 | -5.24782 | 0.00462 | 5.43783 | | 0.00 | 0 | 0 |
| **Work loss days, liver 1st yr 56 -62** | -1.72803 | -5.24782 | 0.00462 | 5.43783 | | -1.72803 | -5.24782 | 0.00462 | 5.43783 | | 0.00 | 0 | 0 |
| **Population norms, 80+** | -1.72803 | -5.79431 | 0.00462 | 4.54008 | | -1.72803 | -5.79431 | 0.00462 | 4.54008 | | 0.00 | 0 | 0 |
| **Population norms, 70-79** | -1.72803 | -5.24782 | 0.00462 | 5.43783 | | -1.72803 | -5.24782 | 0.00462 | 5.43783 | | 0.00 | 0 | 0 |
| **Persons with liver transplant** | -1.72803 | -5.24782 | 0.004624 | 5.43783 | | -1.72803 | -5.24782 | 0.004615 | 5.43783 | | 0.00 | 0 | 9.47E-06 |
| **Persons with icteric hepatitis A** | -1.72803 | -5.24782 | 0.004894 | 5.43783 | | -1.72803 | -5.24782 | 0.004306 | 5.43783 | | 0.00 | 0 | 0.000588 |
| **Persons with anicteric hepatitis A** | -1.72803 | -5.24782 | 0.005033 | 5.43783 | | -1.72803 | -5.24782 | 0.004247 | 5.43783 | | 0.00 | 0 | 0.000785 |
