# Supplementary material for: Correction to: Public health impact and cost effectiveness of routine childhood vaccination for hepatitis a in Jordan: a dynamic model approach
Source: BMC Infect Dis. 2019 Oct 28;19:904. doi: 10.1186/s12879-019-4533-y (PMC6819599; doi:10.1186/s12879-019-4533-y)
Supplement: Supplementary file 1 — Additional file 1. Updated Results (Correction Results Details). [file 12879_2019_4533_MOESM1_ESM.docx]

**Updated Result Details: Public health impact and cost effectiveness of routine childhood vaccination for hepatitis a in Jordan: a dynamic model approach**

We applied the two corrections discussed in the correction note. This document provides additional detailed results and comparison with the original results. Tables 1 and 2 correspond to results presented in Tables 2 and 3 of the original publication. The tables include the results from the original publication (“old”) and the results from corrected model (“new”).

Table 1: Cumulative cases avoided over 5, 10, 25, and 50 years of routine vaccination vs. no vaccine corresponding to Table 2 of the original publication.

| Health Outcome | 5 y | | 10 y | | 25 y | | 50 y | |
| --- | --- | --- | --- | --- | --- | --- | --- | --- |
|  | old | new | old | new | old | new | old | new |
| Any Infection | 326,900 | 584,100 | 748,610 | 1,333,200 | 1,618,100 | 3,098,700 | 4,260,600 | 11,112,200 |
| Asymptomatic | 225,770 | 453,700 | 509,770 | 1,027,900 | 1,085,500 | 2,371,800 | 2,813,200 | 8,452,500 |
| Symptomatic | 101,100 | 130,400 | 238,800 | 305,400 | 532,600 | 727,000 | 1,447,000 | 2,659,800 |
| Outpatient | 99,600 | 129,000 | 235,000 | 302,100 | 524,000 | 718,900 | 1,420,000 | 2,630,100 |
| Hospitalizations | 1,522 | 1,381 | 3,633 | 3,267 | 8,194 | 7,857 | 22,470 | 28,989 |
| Fulminant cases | 24 | 18 | 61 | 45 | 150 | 115 | 510 | 461 |
| Liver Transplants | 5 | 0 | 10 | 2 | 30 | 8 | 90 | 47 |
| Deaths | 4 | 3 | 9 | 7 | 20 | 17 | 80 | 68 |

Table 2: Cumulative economic outcomes per population over 5, 10, 25, and 50 years of routine vaccination vs. no vaccine corresponding to Table 3 of the original publication.

|  | 5 Years | | 10 Years | | 25 Years | | 50 years | |
| --- | --- | --- | --- | --- | --- | --- | --- | --- |
| Economic Outcome | old | new | old | new | old | new | old | new |
| QALY's Gained | 4,800 | 7,719 | 10,521 | 16,659 | 24,144 | 41,745 | 37,502 | 76,699 |
| Vaccination Cost (million $) | 9.40 | 23.78 | 17.52 | 46.42 | 35.76 | 108.08 | 52.83 | 176.58 |
| Disease Cost Avoided (million $) | 8.06 | 9.57 | 18.60 | 21.42 | 47.78 | 57.55 | 81.41 | 111.35 |
| Indirect cost avoided (million $) | 0.99 | 0.54 | 2.61 | 1.43 | 8.00 | 5.00 | 14.03 | 10.60 |
| Total Cost Saved | – | - | 3.69 | - | 20.03 | - | 42.60 |  |
| Cost/QALY | 281.00 | 1,511.74 | Cost saving | 1,180.95 | Cost saving | 873.31 | Cost saving | 619.61 |

Figure 1 updates Figure 3 from the original publication showing the PSA results. The PSA result shows the mean incremental cost effectiveness ratio of $609.68/QALY with no probability of being over the willingness-to-pay threshold of $3600/QALY and about an 8% probability of being cost-saving.


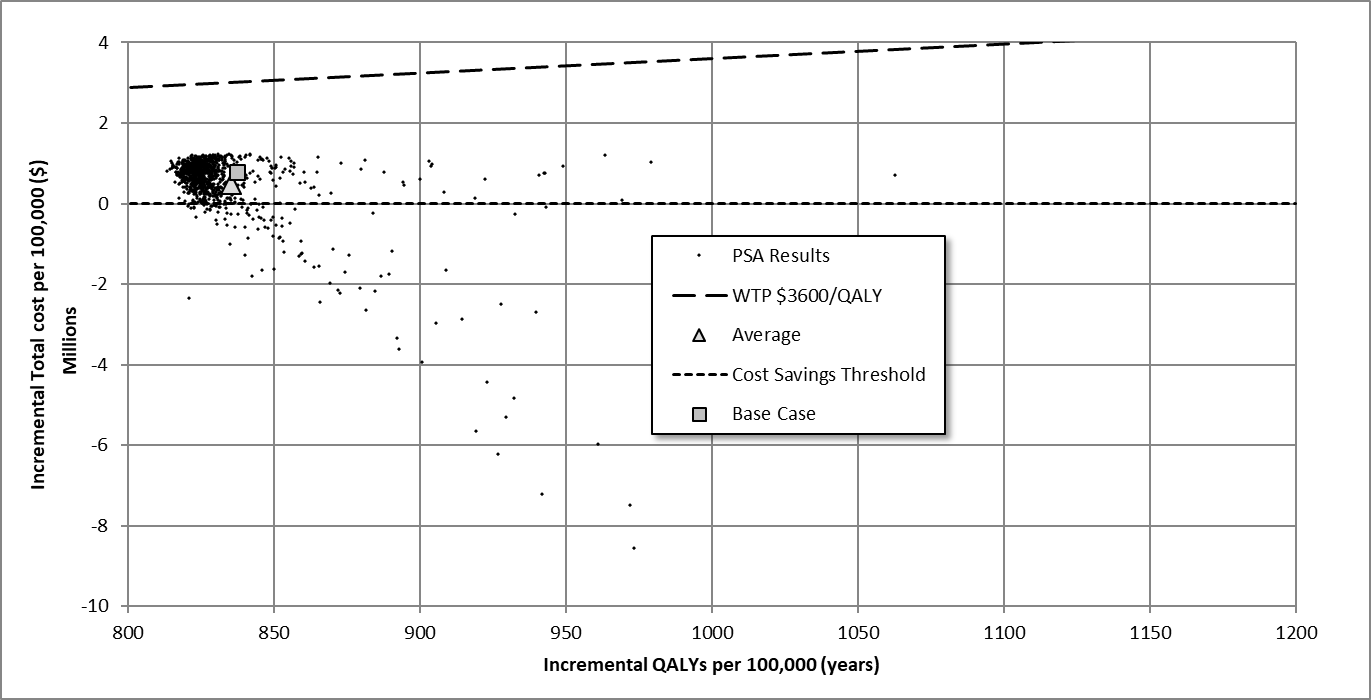


Figure 1: Corrected Probabilistic sensitivity analysis (PSA) results updates Figure 3 from the original publication. Scatter plot of the estimated joint density of incremental total costs and QALYs per 100,000 persons for vaccination program compared to no vaccination. The long-dashed line represents the $3600 WTP threshold above which the program would not be very cost effective. The short-dashed line represents the cost saving threshold below which results are cost-saving. Each black dot represents the resulting incremental cost and effectiveness of one set of parameters from the PSA. The triangle indicates the result corresponding to the average incremental cost and effectiveness. And the square represents the cost-effectiveness of the base case parameter set. All points with negative incremental cost represent cost-saving parameter sets
