# Supplementary material for: Correction to: Public health impact and cost effectiveness of routine childhood vaccination for hepatitis a in Jordan: a dynamic model approach
Source: BMC Infect Dis. 2019 Oct 28;19:904. doi: 10.1186/s12879-019-4533-y (PMC6819599; doi:10.1186/s12879-019-4533-y)
Supplement: Supplementary file 2 — Additional file 2. Updated Results (Updated Tables). [file 12879_2019_4533_MOESM2_ESM.docx]

Table 2 – HAV cases avoided

Cumulative HAV cases avoided for the population of Jordan after 5, 10, 25, and 50 years of universal vaccination.

| Cumulative events avoided with vaccination | Universal Vaccination | | | |
| --- | --- | --- | --- | --- |
|  | 5 years | 10 years | 25 years | 50 years |
| Any Infection | 584,100 | 1,333,200 | 3,098,700 | 11,112,200 |
| Asymptomatic | 453,700 | 1,027,900 | 2,371,800 | 8,452,500 |
| Symptomatic | 130,400 | 305,400 | 727,000 | 2,659,800 |
| Outpatient | 129,000 | 302,100 | 718,900 | 2,630,100 |
| Hospitalizations | 1,381 | 3,267 | 7,857 | 28,989 |
| Fulminant cases | 18 | 45 | 115 | 461 |
| Liver Transplants | 0 | 2 | 8 | 47 |
| Deaths | 3 | 7 | 17 | 68 |

Table 3 – Cost effectiveness

Cumulative cost effectiveness/savings after 5, 10, 25, and 50 years of vaccination program. Cost savings is achieved within 6 years considering indirect costs and within 8 years if indirect costs are excluded. Values are for the entire population.

| Cumulative outcomes | Universal Vaccination | | | |
| --- | --- | --- | --- | --- |
|  | 5 y | 10 y | 25 y | 50 y |
| QALY's Gained | 7,719 | 16,659 | 41,745 | 76,699 |
| Vaccination Cost (millions) | $23.78 | $46.42 | $108.08 | $192.76 |
| Disease Cost Avoided (millions) | $9.57 | $21.42 | $57.55 | $111.35 |
| Indirect cost avoided (millions) | $0.54 | $1.43 | $5.00 | $10.60 |
| Total Cost Savings(millions, including indirect costs) | - | - | - | - |
| Total Cost Savings(millions, excluding indirect cost) | 1,770 | 1,415 | 1,091 | 923 |
| ICER ($/QALY, including indirect costs) | 7,719 | 16,659 | 41,745 | 76,699 |
| ICER ($/QALY, excluding indirect cost) | $23.78 | $46.42 | $108.08 | $192.76 |

QALY, quality-adjusted life-year

ICER, incremental cost-effectiveness ratio
